# Supplementary material for: α-Glucosidase Inhibitory and Antimicrobial Benzoylphloroglucinols from Garcinia schomburgakiana Fruits: In Vitro and In Silico Studies
Source: Molecules. 2022 Apr 15;27(8):2574. doi: 10.3390/molecules27082574 (PMC9032663; doi:10.3390/molecules27082574)
Supplement: Supplementary file 1 [file molecules-27-02574-s001.zip › molecules-1668637-supplementary.pdf]

α-Glucosidase Inhibitory and Antimicrobial Benzoylphloroglucinols from *Garcinia schomburgakiana* Fruits: In vitro and In silico Studies

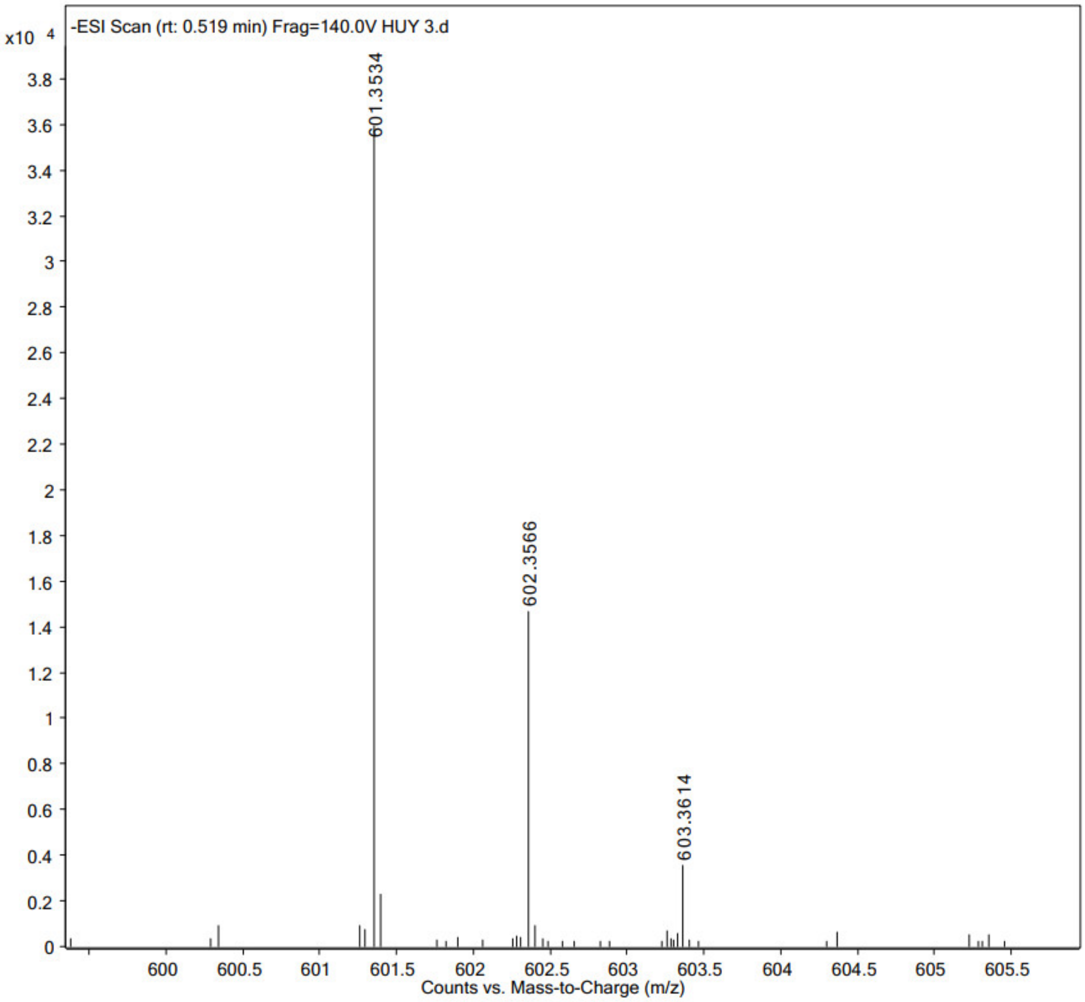

Figure S1. HRESIMS of **1**

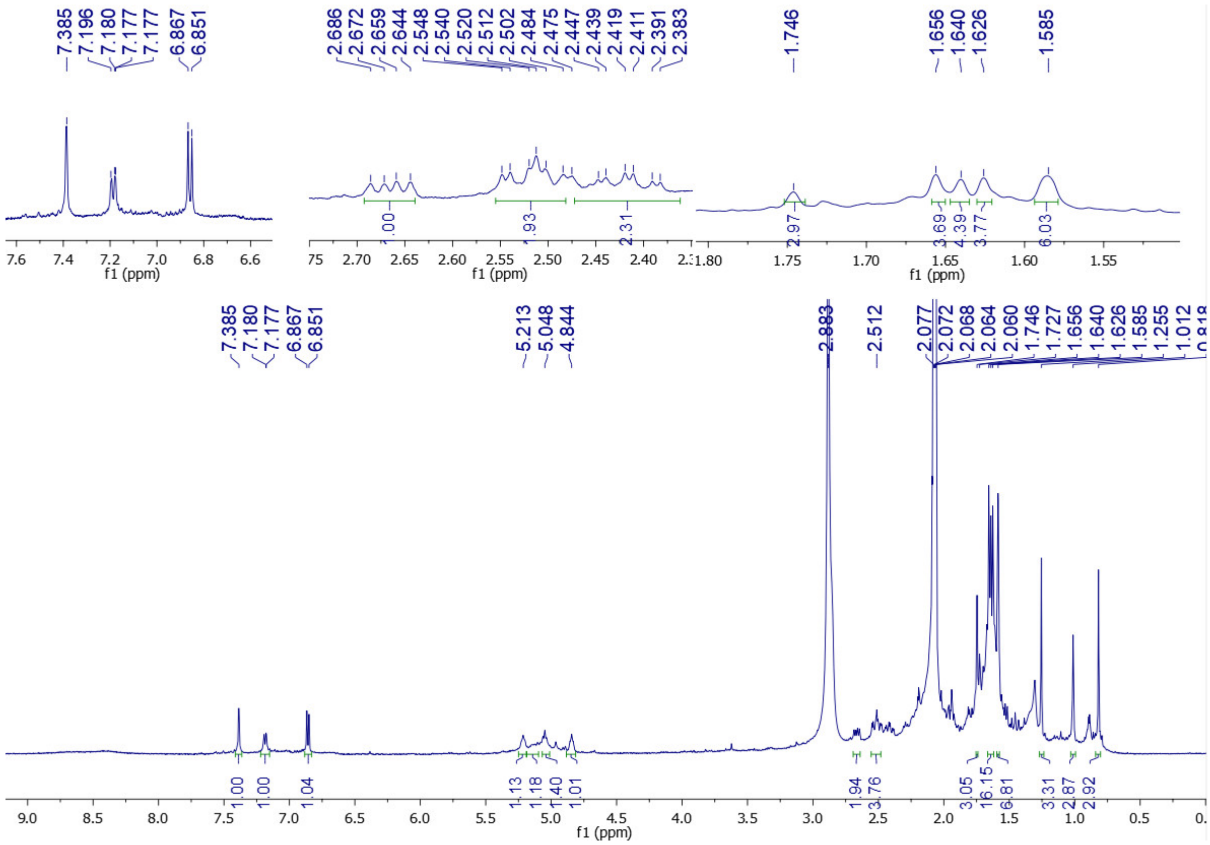

Figure S2. The ^{1}H NMR spectrum of **1** in acetone-d_6.

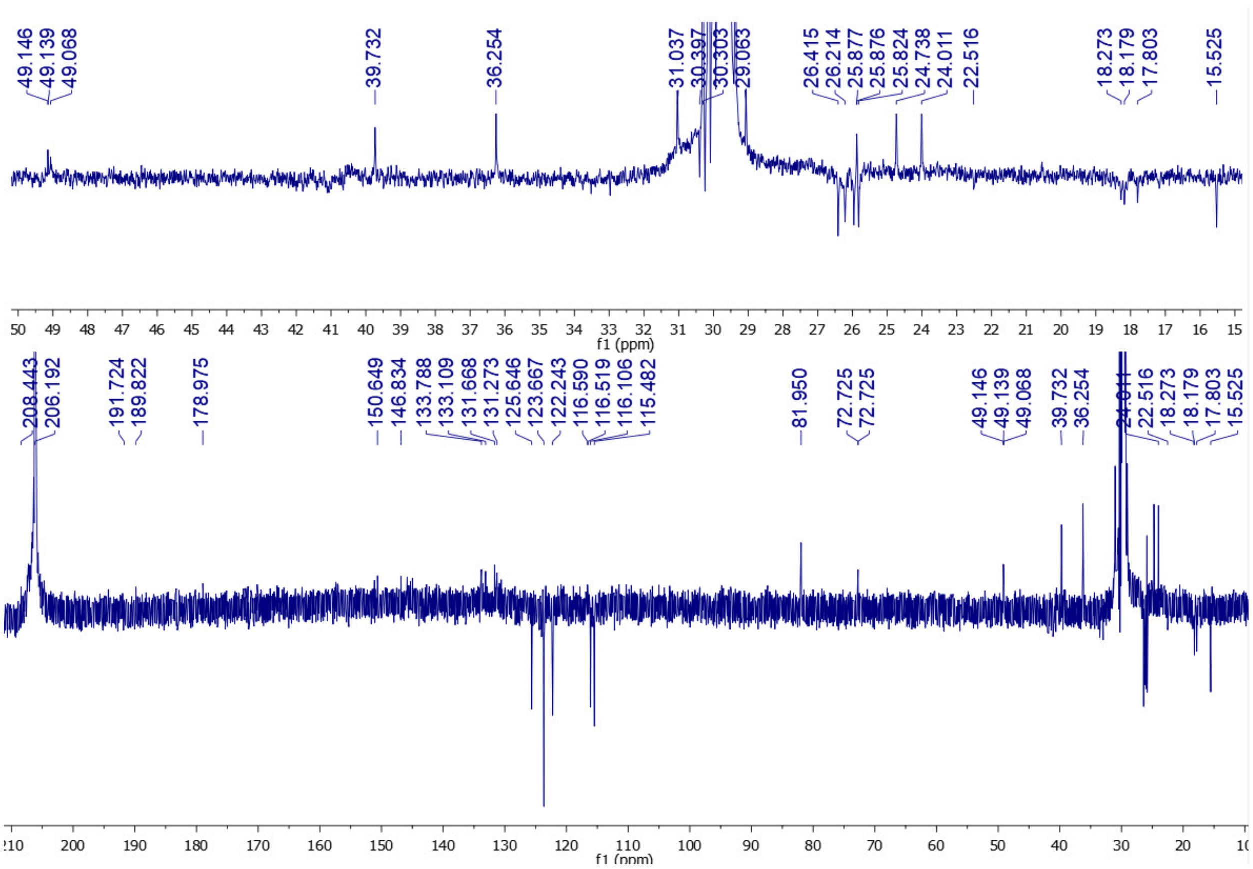

Figure S3. The ^{13}C NMR spectrum of **1** in acetone-d_6.

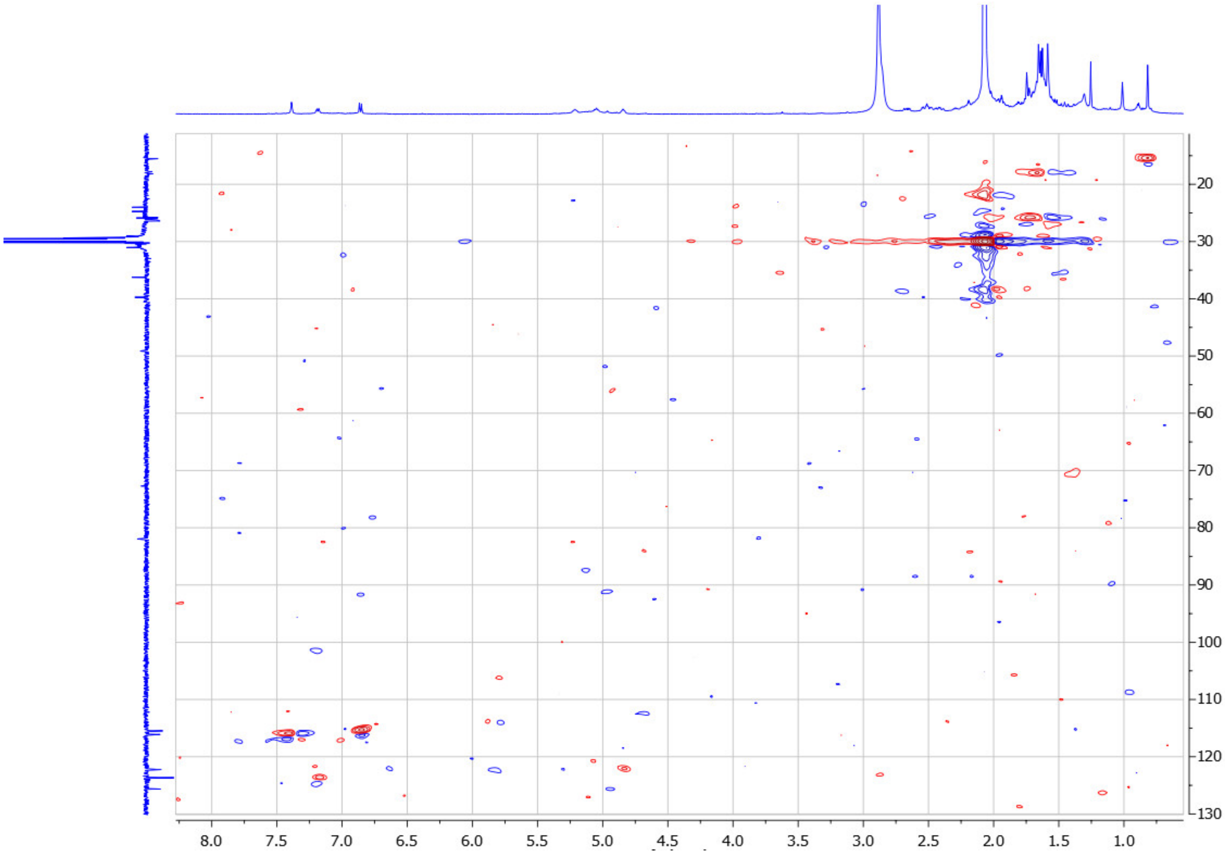

Figure S4. The HSQC spectrum of **1** in acetone-d_6.

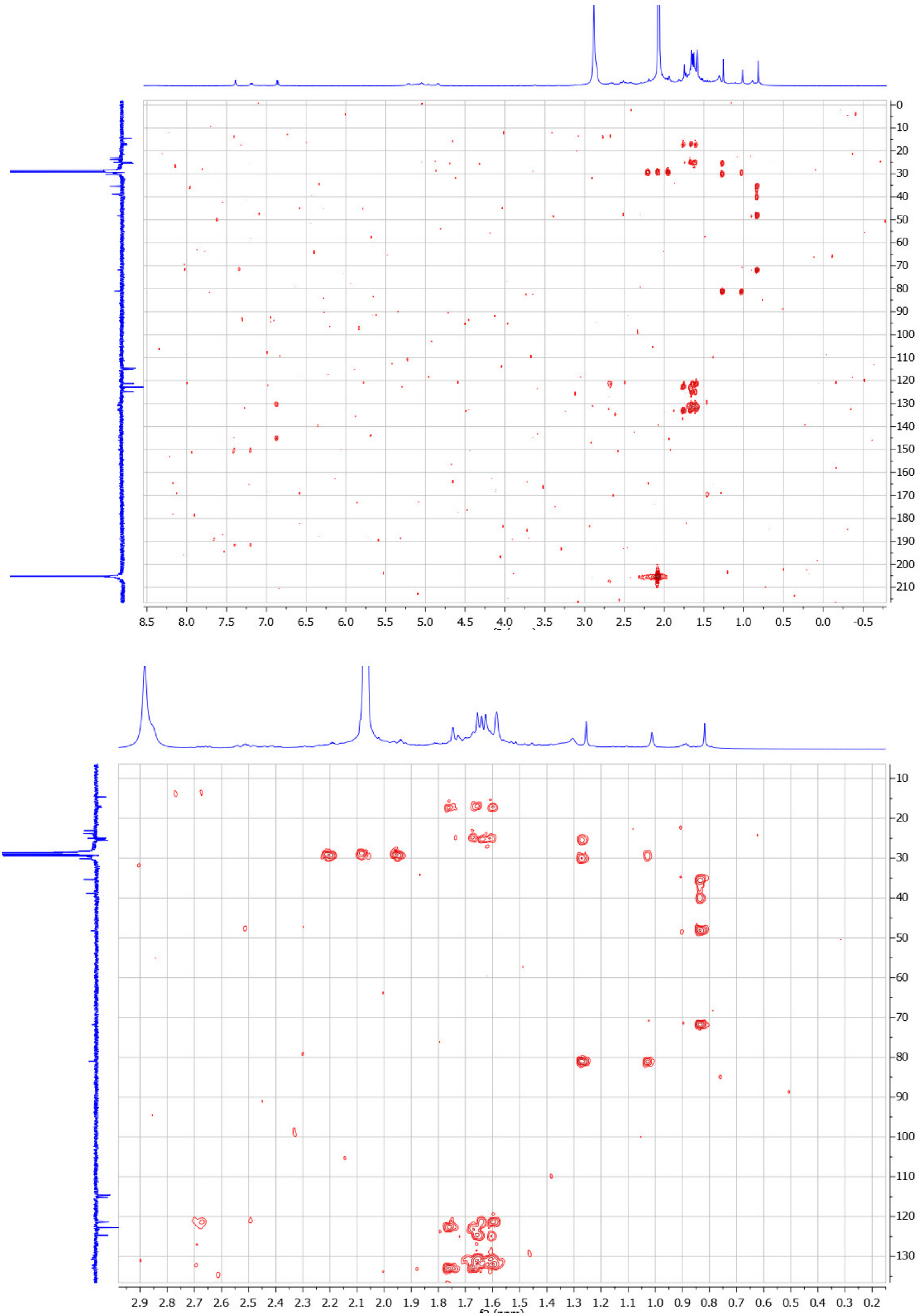

Figure S5. The HMBC spectrum of **1** in acetone-d_6.

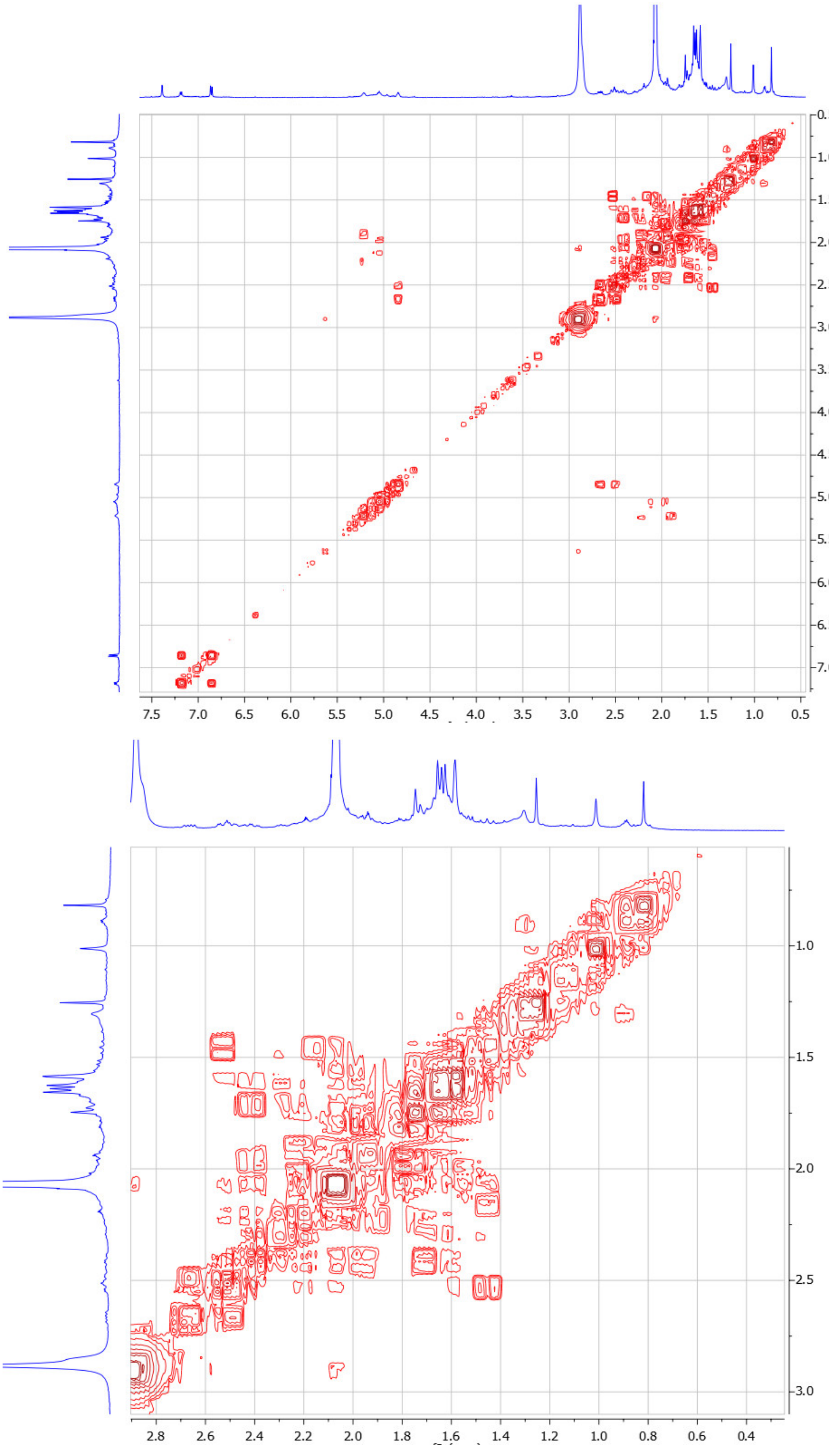

Figure S6. The COSY spectrum of **1** in acetone-d_6.

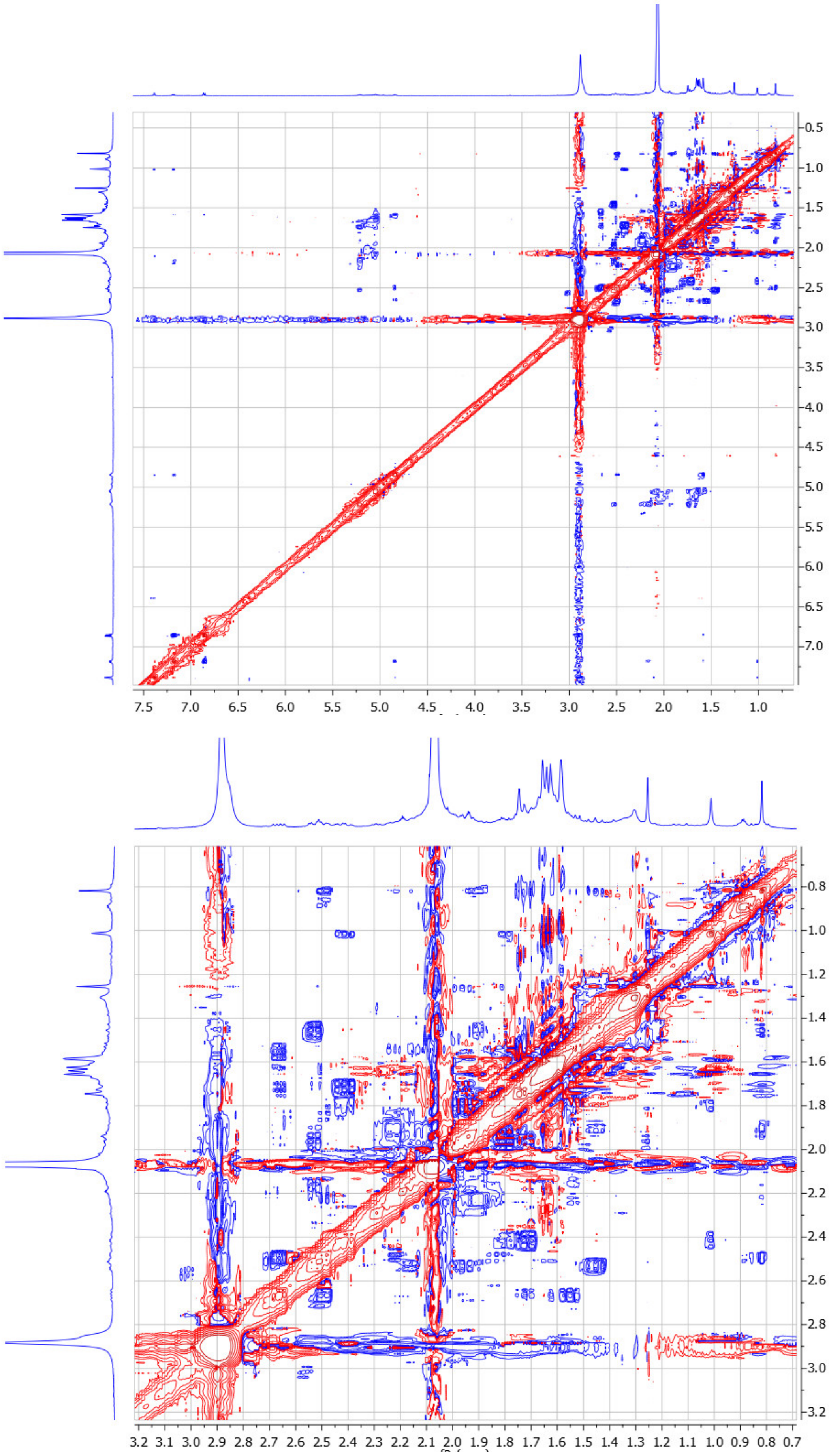

Figure S7. The NOESY spectrum of **1** in acetone-d_6.

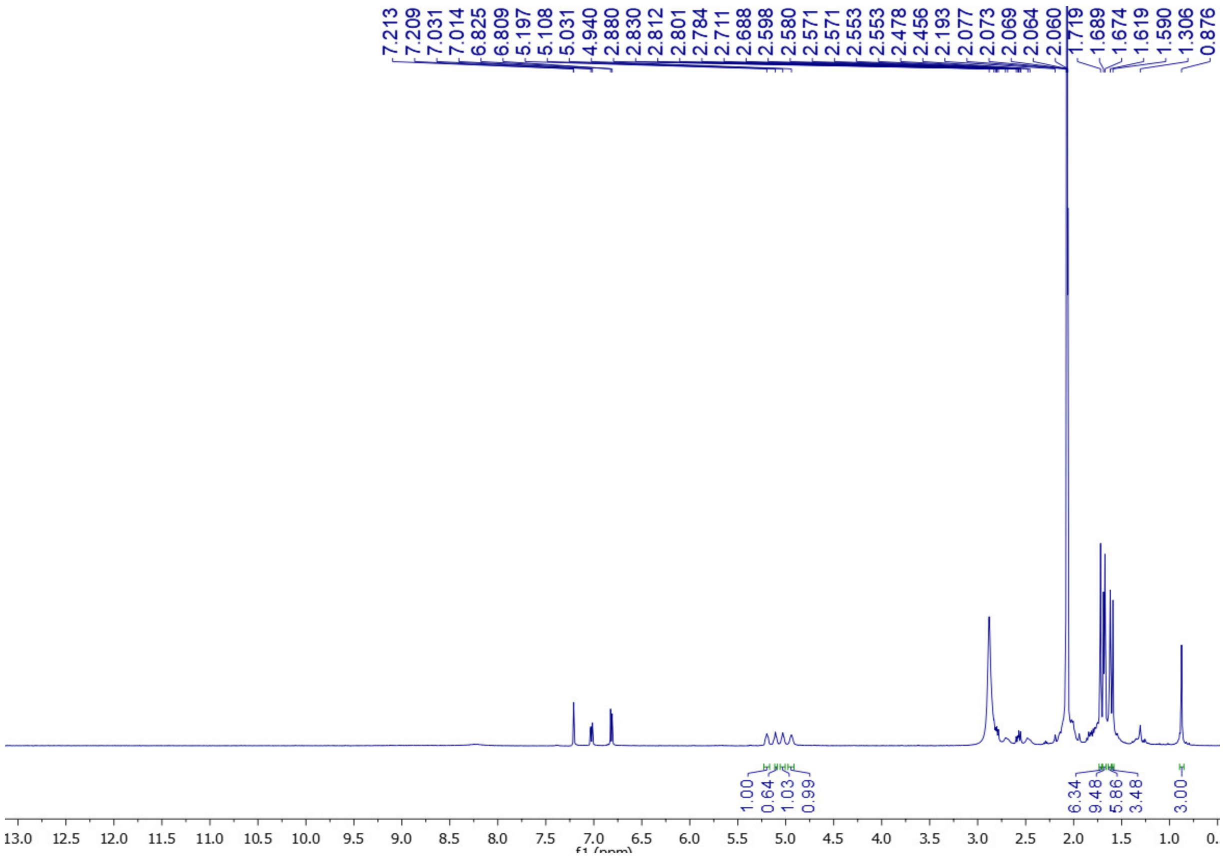

Figure S8. The ^{1}H NMR spectrum of **2** in acetone-d_6.

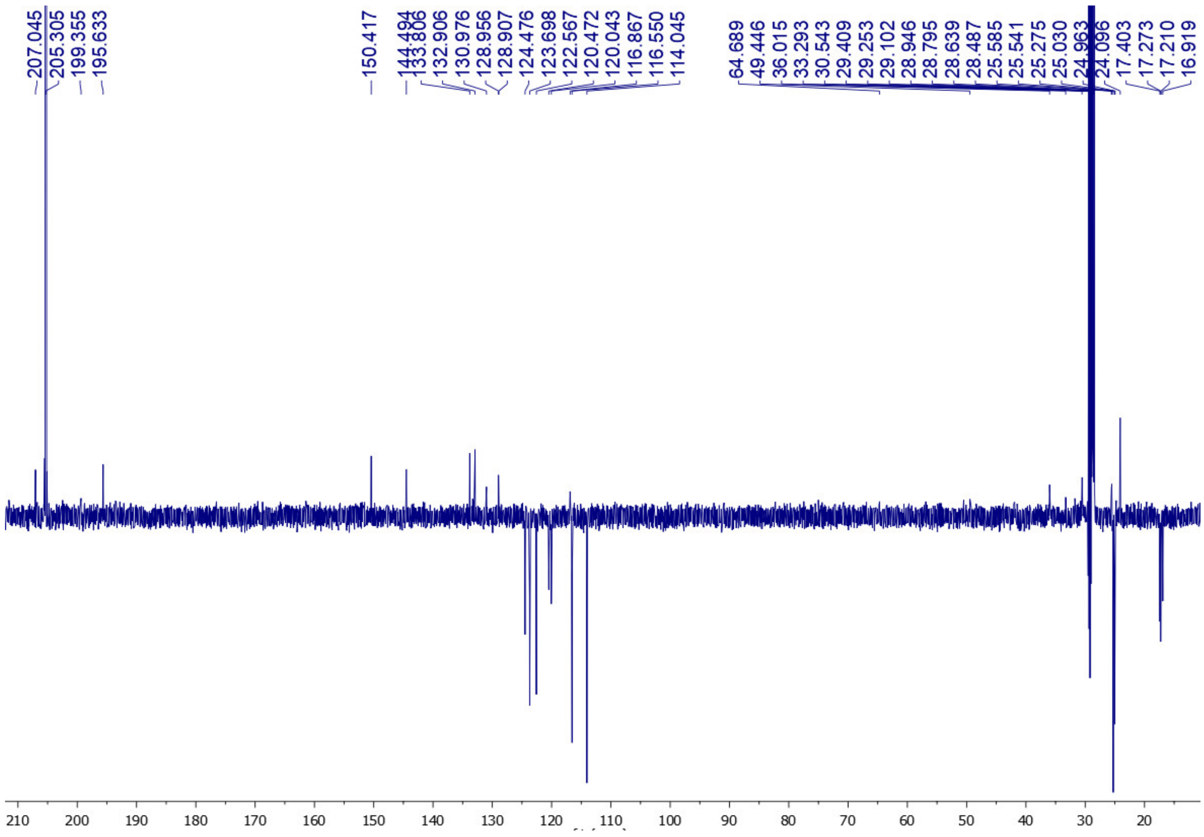

Figure S9. The ^{13}C NMR spectrum of **2** in acetone-d_6.

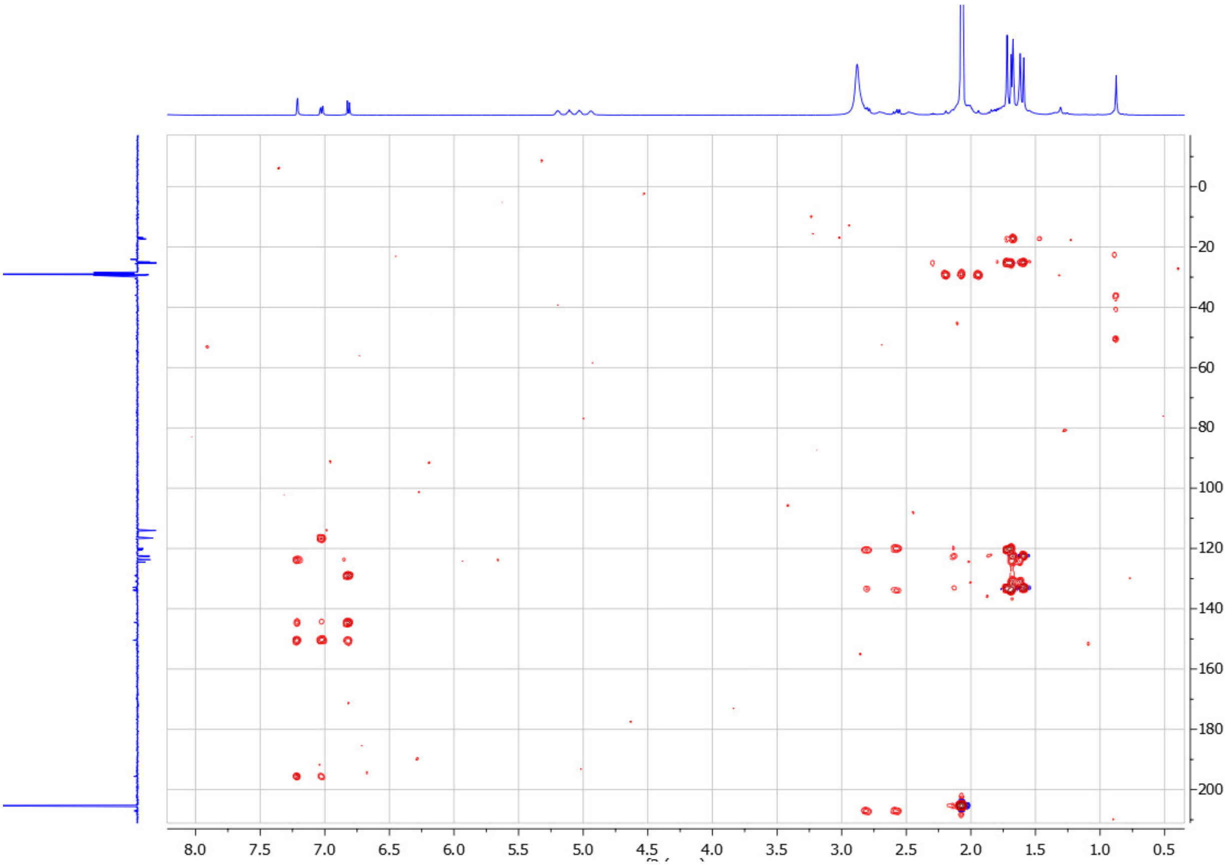

Figure S10. The HMBC spectrum of **2** in acetone-d_6.

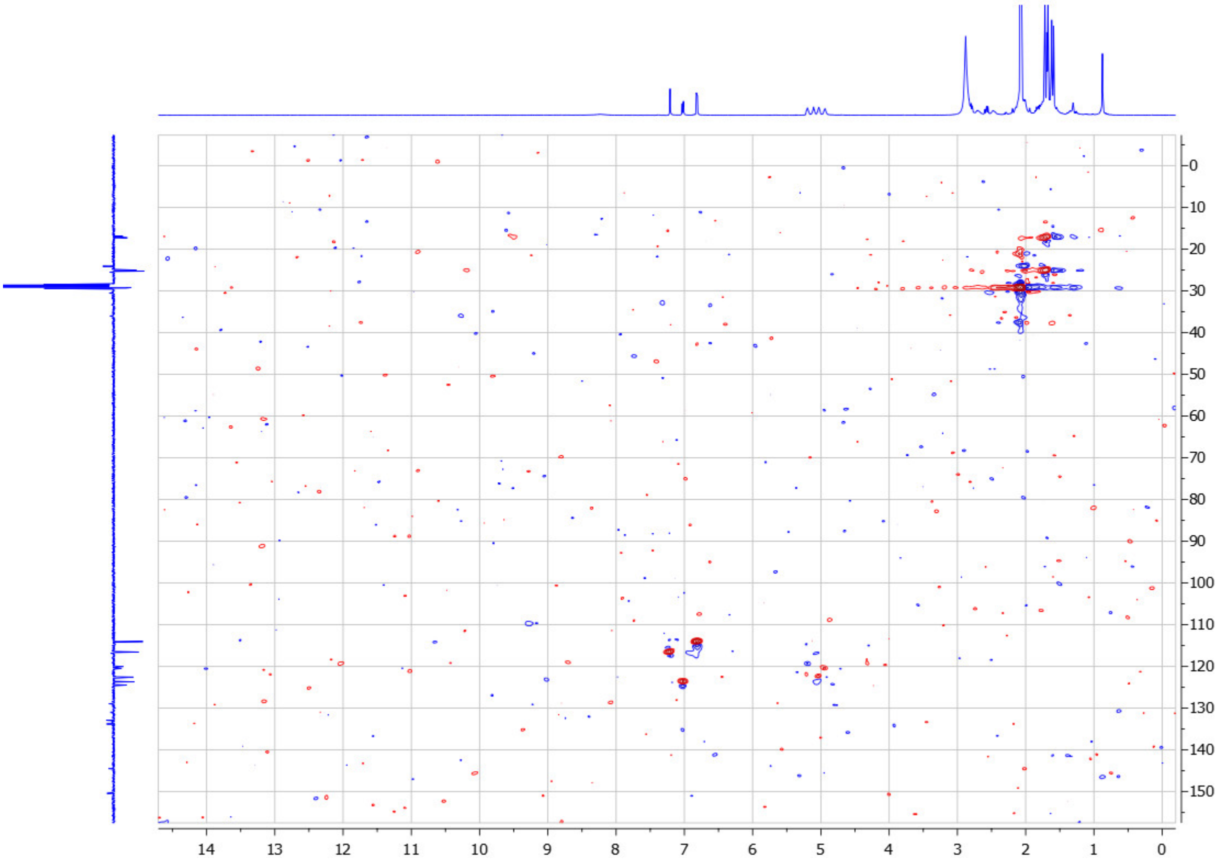

Figure S11. The HSQC spectrum of **2** in acetone-d_6.

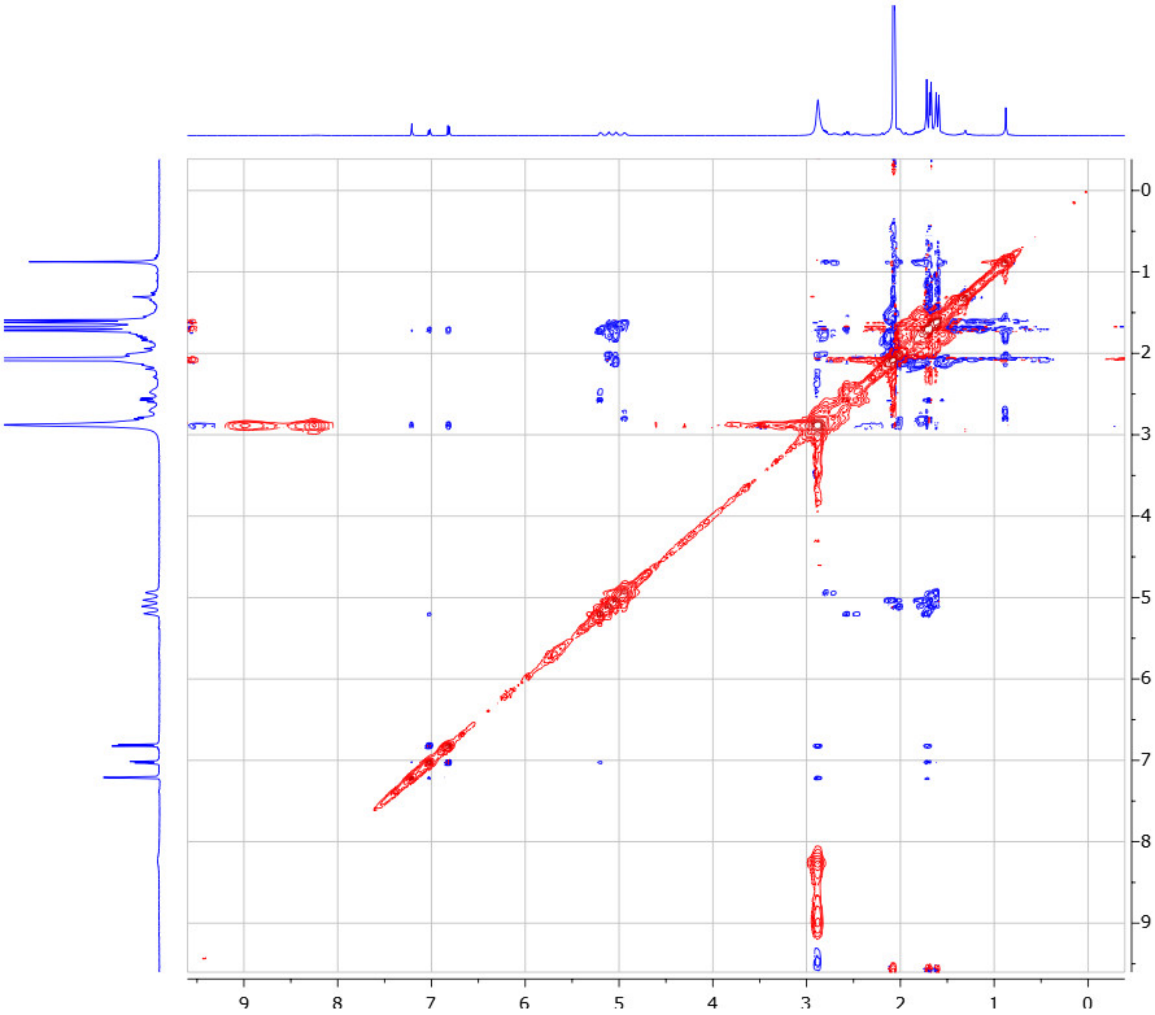

Figure S12. The NOESY spectrum of **2** in acetone-d_6.

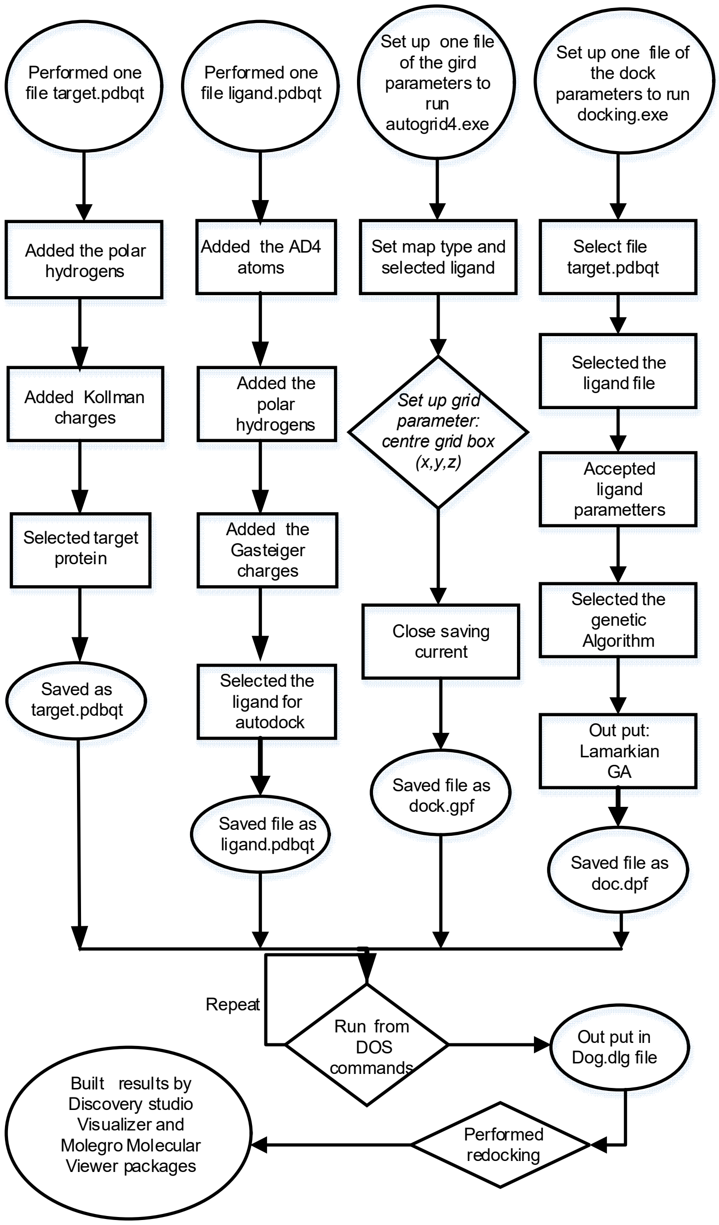

Scheme S1. The general Procedure docking of the most stable conformation ligand to the receptor.
